# Complete Genome Sequence of *emm*1 *Streptococcus pyogenes* A20, a Strain with an Intact Two-Component System, CovRS, Isolated from a Patient with Necrotizing Fasciitis

**DOI:** 10.1128/genomeA.00149-12

**Published:** 2013-02-07

**Authors:** Po-Xing Zheng, Kun-Ta Chung, Chuan Chiang-Ni, Shu-Ying Wang, Pei-Jane Tsai, Woei-Jer Chuang, Yee-Shin Lin, Ching-Chuan Liu, Jiunn-Jong Wu

**Affiliations:** a Institutes of Basic Medical Sciences; b Molecular Medicine; c Departments of Medical Laboratory Science and Biotechnology; d Microbiology and Immunology; e Biochemistry and Molecular Biology; f Pediatrics, College of Medicine, National Cheng Kung University, Tainan City, Taiwan; g Center of Infectious Disease and Signaling Research, National Cheng Kung University, Tainan City, Taiwan

## Abstract

Here, we announce the complete sequence of *Streptococcus pyogenes* A20. This strain was isolated from a patient with necrotizing fasciitis. Given that A20 harbors an intact two-component system, CovRS, the discovery of its genome sequence provides more insight into the pathogenesis of a pandemic *emm*1 strain.

## GENOME ANNOUNCEMENT

*Streptococcus pyogenes* is an important human pathogen that causes many diseases, ranging from sore throat to life-threatening necrotizing fasciitis (1). The serotype *emm*1 reemerged and caused a pandemic infection after 1980 (2). Recent studies demonstrated that prophage integration and the acquisition of new phage-encoded virulence factors caused increased disease severity in *emm*1 *S. pyogenes* (3). The mutation of the two-component system CovRS, with reduced expression of the cysteine protease SpeB, is important in the development of severe infections, and almost 46.3% of invasive strains harbor a mutated *covRS* gene (4, 5). The pathogenesis of SpeB^−^/CovRS mutant *emm*1 *S. pyogenes* has been addressed extensively (6, 7). However, 50% of invasive strains have wild-type *covRS*, and the pathogenic mechanism of SpeB^+^/CovRS wild-type *emm*1 *S. pyogenes* remains to be determined.

*S. pyogenes* strain A20 was isolated from a blood sample from a patient with necrotizing fasciitis. A20 is an *emm*1/sequence type (ST)28 strain, in which CovRS is intact and SpeB is highly expressed (8); this promotes internalization into (and the apoptosis of) epithelial cells (9, 10). A20 also induces high mortality in BALB/c mice (11), which facilitates *in vivo* studies of its pathogenesis. Therefore, A20 was chosen here for sequence analysis.

The *S. pyogenes* strain A20 genome was sequenced with an Illumina Genome Analyzer IIx (Illumina, CA). Library construction, sequencing, base calling, and *de novo* assembly were performed in Yourgene Bioscience (Taipei, Taiwan). Briefly, a paired-end library was constructed with an average distance of 300 bp. Base calling was performed by the Genome Analyzer systems software 1.5. A total of 37,148,070 high-quality reads (2 × 75 bp) were obtained, which provided almost 1,400-fold coverage of the genome. *De novo* assembly was performed with the CLC Genomics Workbench (CLCbio, Aarhus, Denmark), and a total of 31 contigs were obtained. Gaps were filled by Sanger sequencing (Mission Biotech, Taipei, Taiwan). The coding regions were analyzed and annotated using the CLC Genomics Workbench. The prophages were identified using the PHAge Search Tool (PHAST) (12).

The *S. pyogenes* strain A20 harbored a single circular genome of 1,837,281 bp, with an average G+C content of 38.54%. There were 1,828 open reading frames, 67 tRNA genes, and 18 rRNA genes. Three putative prophages were identified, and several phage-encoded virulence factors were found, including superantigen, streptodornase, and mitogenic factors.

### Nucleotide sequence accession number.

The complete whole genome sequence of *S. pyogenes* strain A20 has been deposited in the NCBI under the accession no. CP003901 (GenomeProject no. SUB130559).
